# A new tool to evaluate burnout: the Italian version of the BAT for Italian healthcare workers

**DOI:** 10.1186/s12889-022-12881-y

**Published:** 2022-03-09

**Authors:** Ivan Borrelli, Paolo Emilio Santoro, Caterina Fiorilli, Giacomo Angelini, Ilaria Buonomo, Paula Benevene, Luciano Romano, Maria Rosaria Gualano, Carlotta Amantea, Umberto Moscato

**Affiliations:** 1 Department of Life Sciences and Public Health, Catholic University of the Sacred Heart, Rome, Italy; 2 Department of Women, Children and Public Health Sciences, Fondazione Policlinico Universitario Agostino Gemelli IRCCS, Rome, Italy; 3 Department of Human Sciences, University of LUMSA, Rome, Italy; 4 Department of Human Sciences, European University of Rome, Rome, Italy; 5 Department of Public Health, University of Turin, Turin, Italy

**Keywords:** Burnout, Psychological distress, Psychosomatic, Occupational, Questionnaire, Validation, Tool, Healthcare workers

## Abstract

**Background:**

Healthcare workers (HCWs) represents one of the highest-risk population to develop burnout symptoms. Recently, a new tool has been designed to measure several dimensions that capture an exhaustive expression of burnout symptoms by six dimensions (i.e., exhaustion; mental distance; cognitive impairment; emotional impairment; psychological distress; psychosomatic complaints).

**Methods:**

The current study aims to adapt the Burnout Assessment Tool (BAT) to an Italian Healthcare workers’ sample confirming the original second-order factorial structure. Furthermore, we expected to find good indexes of reliability and validity tests. Participants were 697 Italian Health Care Workers (Female = 68.44%; mean age = 36.47; SD = 11.20). Data were collected by self-report questionnaires submitted by the snowball method.

**Results:**

Findings show a good fit of the BAT’s structure, confirming the hypothesized second-order factorial model. Furthermore, good reliability has been established with the study’s measures.

**Conclusions:**

The BAT for HCWs is eligible as a new tool to evaluate burnout in the at-risk HCWs as a multi-facet constellation of symptoms.

## Background

In the earlier introduction of the burnout concept, Freudenberger (1974) describes the syndrome as a state of mind characterized by feeling inadequate, wearing out, and by a sense of exhaustion. These conditions are due to excessive demands on vigor, strength, and personal resources, which come from continuing pressures of working with emotionally needy and demanding individuals [1]. Decades of research on burnout led scholars to assume it involves mental and physical exhaustion, which increases with prolonged exposure to work-related troubles [2]. In other words, burnout arises when workers continuously have to cope with chronic emotional and interpersonal stressors due to a challenging work environment. It comprises three psychological states: emotional exhaustion, cynicism, and decreased professional accomplishment [3–5]. When people feel burned out, they are more sensitive to stressful events, which, in turn, may trigger a mechanism of reaction (i.e., aggressive behaviors) or detachment (i.e., depersonalization and cynicism). More common symptoms are high blood pressure, heart disease, sleep problems, backache, obesity, diabetes. Frequently, burned-out workers manifest irritability, anger, and general depression, which may detach from the social context of the work environment.

Interestingly, burnout is a kind of professional illness strictly linked to the workers’ motivation, engagement, and self-efficacy [6, 7]. Finally, several findings support the high correlation between burnout and reduced cognitive functioning [8]. In sum, burnout involves several emotional, cognitive, and behavioral expressions, which may lead to profoundly considering the dramatic effect of burnout on the quality of human services provided by institutions like schools and hospitals.

Medical professionals experience several sources of stress (e.g., human suffering, death, workload pressure), which are the central core of their daily work life [9, 10]. Effectively, healthcare workers (HCWs) are particularly exposed to the risk of professional illness, such as burnout. Recently Hall and colleagues [11] have reviewed studies addressing the adverse consequences of HCWs’ burnout state. Interestingly, the more doctors show burnout symptoms, the more poor medical service [12]. Doctors’ errors are the most critical menace to patients’ safety. The strong association observed in several studies [13–16] among burnout, error, and inefficiency service leads to seriously takes into account this risk: burned-out doctors are more inefficient than their colleagues.

In this regard, preventing and monitoring HCWs’ well-being is of great concern in terms of adverse consequences for both the personal life of professionals and the patient’s care [12, 17]. Several instruments have been used to measure the HCWs’ burnout; for example, the Copenhagen Burnout Inventory [18], the Oldenburg Burnout Inventory [19], and the Pines’ Burnout Measure [20] (for a review, see Shoman et al. [21]). Nevertheless, the Maslach Burnout Instrument represents the golden standard [22]. The MBI comprises three subscales: emotional exhaustion, depersonalization, and reduced personal accomplishment. It is adopted and translated in several languages confirming its three-factors structure with good reliability and validity indexes.

Nowadays, burnout is not yet a clinical diagnosis. The MBI is frequently used, as Maslach and colleagues [23] suggested, to recognize the high level of burnout, particularly in the environments devoted to human resources management [24–26]. In this regard, the instrument mainly used to evaluate burnout, the MBI [23], is usually adopted as a unidimensional instrument cause of abundant findings confirming the prominence of emotional exhaustion dimensions describing burnout toward MBI. There is an ongoing debate regarding how and whether the three subscales of MBI well capture the pain. For example, the level of personal accomplishment (i.e., high or low level) does not clearly describe the burnout state [25, 27, 28].

Even if the discussion about burnout construct’s multi-dimensionality or one-dimensionality is far from closing, a new tool has been recently proposed, expected to overcome some limitations of previous burnout measures. Schaufeli et al. [29] have recently introduced a new device, the Burnout Assessment Tool (BAT); it is a multi-factors tool where several dimensions are considered part of burnout suffering and its externalize and internalize expressions. The BAT is already adopted in the Italian language, demonstrating good reliability and validity [30, 31], encouraging its extensive use in burnout measures. However, no previous research has adopted BAT’s measures to an HCW’s sample to our knowledge.

Bringing together the literature mentioned above, the central core of the current study was to adapt the Italian version of the Burnout Assessment Tool (BAT) with a sample of Health Care Workers.

## Method

### Aim and hypotheses

The general objective of this study was to adapt the Italian version of the Burnout Assessment Tool (BAT [29]) for an HCWs’ sample. Expressly, given the solid theoretical construct and earlier Italian validation of the BAT (for the general population and a teachers’ sample, respectively Consiglio et al. [31] and Angelini et al. [30]), we set the following aims and hypotheses. We expected: 1) to confirm the second-order factorial structure of the HCW’s version of the BAT (BATxHCW); 2) to obtain good fit indexes in the six-factor model; 3) to confirm the internal reliability of the BATxHCW; 4) to obtain good internal and external validity of the BATxHCW when correlated with the emotional exhaustion subscale of the MBI, with the World Health Organization Well-Being Index (WHO-5), and finally, with the SF-12 Health Survey (SF-12).

## Participants

The study involved 697 Italian Health Care Workers of an age range of 21 to 73 years (M age = 36.47 years, SD = 11.20), of whom 220 were men (31.56%), 477 were women (68.44%). The inclusion criteria in the study were that the participants were Italian workers that operated in the Health Care sector and voluntarily agreed to participate.

## Procedure

The cross-sectional study was carried out in Italy between May and June 2021 using a snowball technique implemented among workers of hospital institutions. The data was collected online via the G Suite Google Platform, which did not allow the procedures if the fields were not filled in. Therefore no survey has been compiled with missing. The hospital mailing list recruited HCWs who were invited to participate in a survey. Participants were informed of the research objectives and were assured that their responses remained anonymous and no personal data was acquired or kept. The research project was approved by the Ethics Committee for Scientific Research (CERS) of LUMSA University, and the study was conducted under the Declaration of Helsinki. This study was conducted under the requirements of privacy and informed consent laid down by current Italian law (Law Decree DL-196/2003).

## Measures

Burnout Assessment Tool (BAT original version [29], Italian version [30]; BAT-C’s α = 0.924), including 33 items on a five-point Likert scale, ranging from “never” (1) to “always” (5). The BAT comprises four core dimensions of burnout (i.e., exhaustion, mental distance, cognitive and emotional impairment). Furthermore, two dimensions refer to the secondary symptoms of burnout (i.e., psychological distress and psychosomatic complaints). Finally, the BAT offers a sub-total score for each subscale and an overall burnout score.

Maslach Burnout Inventory-General Survey (MBI-GS original version [32], Italian version [33]) includes 22 items on a seven-point Likert scale ranging from “never” (0) to “every day” (6). The MBI-GS comprises three dimensions (i.e., emotional exhaustion, EE; depersonalization, DP; and personal accomplishment, PA). In this study, only the 9-item Exhaustion scale was used to assess the feeling of being exhausted by the job. In this study, Cronbach’s alpha was α = 0.93.

World Health Organization Well-Being Index (WHO-5 original version [34], Italian version [35]) consisting of 5 items on a six-point scale, from “all of the time” (0) to “at no time “(5). In this study, Cronbach’s alpha was α = 0.88.

Health Survey Short Form (SF-12 original version [36], Italian version [37]) includes 12 items on a five-point Likert scale with different ranges for each question; the SF-12 comprises two dimensions (i.e., physical aspects and mental aspects). In this study, Cronbach’s alpha was α = 0.85.

## Data analysis

BAT has a solid theoretical construct [29] and is organized in a six-factor structure. A confirmatory factor analysis (CFA) for categorical variables was performed to test the goodness-of-fit of the latent system underlying the indices of Burnout. We used the Root Mean Square Error of Approximation (RMSEA), Comparative Fit Index (CFI), Tucker Lewis Index (TLI), and Standardized Root Mean Square Residual (SRMR) as measures of goodness-of-model fit. We considered fair fit indicators if the RMSEA was less than 0.08, and the CFI, TLI, equal or higher than 0.90 and SRMR of 0.08 is acceptable [38–41]. We evaluated the reliability of the Italian version of the questionnaire and each of its six dimensions using Cronbach’s alpha coefficients of internal consistency. To have a more accurate measure of internal consistency, we used a second index, the McDonald’s Omega. The index is proved to be a more sensitive index of internal consistency [42, 43]; McDonald’s Omega values > 0.8 can be interpreted as good internal reliability [44]. Data analysis was performed with STATA 16 software and R Studio 2021.09.0 Build 351 using the Lavaan package.

## Results

### Factorial Validity

Core Symptoms are measured using 23 items, mean is 51.23 (SD = 15.44), while Secondary Symptoms are calculated using ten items, mean is 23.32 (SD = 7.44). We proceeded with the Second Order Confirmatory Factor Analysis of the 33 item tool and six factors, the results of which are presented in Table 1.

**Table 1:** Second-Order Confirmatory Factor Analysis

	**χ2**	**p**	**df**	**CFI**	**TLI**	**SRMR**	**RMSEA**
BATxHCWs	2647.923	0.00	494	0.93	0.92	0.08	0.08

Indicators of adaptation of the CFA model (root mean square error of approximation RMSEA = 0.08 [90%CI 0.076–0.082], comparative fit index-CFI = 0.93, Tucker-Lewis coefficient-TLI = 0.92) showed a good adaptation of the model of the six-dimensional structure of the 33-item questionnaire. Standardized room means square residual of 0.08—SRMR—is a proper fit.

### Reliability

For analyzing the internal consistency we have calculated Cronbach's α (Cronbach, 1951). The questionnaire had optimal internal consistency reliability, α = 0.94 for the 33-item questionnaire. Internal reliability levels observed in the two subscales were optimal for core symptoms α = 0.94 and good for secondary symptoms 0.85; five of six dimensions of Burnout Assessment Tool was optimal or good (exhaustion α = 90, Mental Distance α = 0.82, Cognitive Impairment α = 0.92, Emotional Impairment α = 0.87, Psychological Distress α = 0.82), and one was discreet (Psychosomatic Complaints α = 0.72) (Table 2).

**Table 2:** Internal consistency of Italian version **Cronbach's α**

Items	Dimensions	Cronbach's α of dimensions	Subscale	Cronbach's α of Subscale	Cronbach's α total of BAT for Health Care Workers
1–8	**Exhaustion**	0.90	**Core Symptoms**	0.94	0.94
9–13	**Mental Distance**	0.82			
14–18	**Cognitive Impairment**	0.92			
19–23	**Emotional Impairment**	0.87			
24–28	**Psycological Distress**	0.82	**Secondary Symptoms**	0.85	
29–33	**Psychosomatic Complaints**	0.72			

We proceeded with McDonald's ω (McDonald, 1999) to analyze the internal consistency using a six-factor second-order CFA; the Second Order Confirmatory Factor Analysis of the 33 item tool and six factors; ω = 0.96 for the 33-item questionnaire. Internal reliability levels observed in the six dimensions of Burnout Assessment Tool was: exhaustion ω = 91, Mental Distance ω = 0.83, Cognitive Impairment ω = 0.82, Emotional Impairment ω = 0.82, Psychological Distress ω = 0.81 and Psychosomatic Complaints α = 0.79. (Table 3).

**Table 3:** Internal consistency of Italian version (McDonald's ω)

Items	Dimensions	McDonald's ω of dimensions	McDonald's ω total of BAT for Health Care Workers
1–8	**Exhaustion**	0.91	0.96
9–13	**Mental Distance**	0.83	
14–18	**Cognitive Impairment**	0.82	
19–23	**Emotional Impairment**	0.82	
24–28	**Psycological Distress**	0.81	
29–33	**Psychosomatic Complaints**	0.79	

### Construct validity

Finally, we calculated the Pearson correlation coefficients among all scales of BAT and the two scales used as convergent and discriminant validity (Table 4).

**Table 4:** Correlations matrix for convergent and discriminant BAT’s validity (*N* = 697)

	**MBI_EE**	**WHO**	**SF-12**
**BAT**	0.803***	-0.626***	0.354***
**BAT_E**	0.772***	-0.556***	0.258***
**BAT_MD**	0.645***	-0.437***	0.227***
**BAT_CI**	0.505***	-0.413***	0.221***
**BAT_EI**	0.586***	-0.370***	0.244***
**BAT_PD**	0.631***	-0.626***	0.343***
**BAT_PC**	0.478***	-0.444***	0.356***

*** *p* < 0.001. *BAT* represents Burnout Assessment Tool (total score), *BAT_E* represents Exhaustion dimension, *BAT_MD* represents Mental Distance dimension, *BAT_CI* represents Cognitive Impairment dimension, *BAT_EI* represents Emotional Impairment dimension, *BAT_PD* represents Psychological Distress dimension, *BAT_PC* represents Psychosomatic Complaints dimension, *MBI_EE* represents Emotional Exhaustion scale of Maslach Burnout Inventory, *SF-12* represents Quality of Life Short Form (total score), *WHO* represents World Health Organization Well-Being Index (total score)

Pearson correlation analysis showed that the MBI Emotional Exhaustion scale was positively correlated with all dimensions of BAT: Exhaustion (*r* = 0.772, *p* ≤ 0.001), Mental distance (*r* = 0.645, *p* ≤ 0.001), Emotional impairment (*r* = 0.505, ≤ 0.001), Cognitive impairment (*r* = 0.586, *p* ≤ 0.001), Psychological distress (*r* = 0.631, *p* ≤ 0.001) and Psychosomatic complaints (*r* = 0.478, *p* ≤ 0.001). Furthermore, the Pearson correlation analysis showed that the total BAT score was positively correlated with Emotional Exhaustion scale of Maslach Burnout Inventory (*r* = 0.803, *p* ≤ 0.001). Regarding the World Health Organization Well-Being Index, the analysis highlighted significant negative correlations with the BAT scores: Exhaustion (*r* = -0.556, *p* ≤ 0.001), Mental distance (*r* = -0.437, *p* ≤ 0.001), Cognitive impairment (*r* = -0.413, *p* ≤ 0.001), Emotional impairment (*r* = -0.370, *p* ≤ 0.001), Psychological distress (*r* = -0.626, *p* ≤ 0.001) and Psychosomatic complaints (*r* = -0.444, *p* ≤ 0.001). Furthermore, the Pearson correlation analysis showed that the WHO was negatively correlated with the total BAT score (*r* = -0.626, *p* ≤ 0.001). Finally, with regards to the Quality of Life Short Form (SF-12), the analysis highlighted significant positive correlations with the BAT scores: Exhaustion (*r* = -0.258, *p* ≤ 0.001), Mental distance (*r* = -0.227, *p* ≤ 0.001), Cognitive impairment (*r* = -0.221, *p* ≤ 0.001), Emotional impairment (*r* = -0.244, *p* ≤ 0.001), Psychological distress (*r* = -0.343, *p* ≤ 0.001) and Psychosomatic complaints (*r* = -0.356, *p* ≤ 0.001). Furthermore, Pearson correlation analysis showed that the SF-12 was positive correlated with total BAT score (*r* = -0.354, *p* ≤ 0.001).

## Discussion

This study aimed to adapt the Italian version of the Burnout Assessment Tool [29] with data gathered from a sample of Italian for HCWs. According to Hiver et al. [45], new procedures and tools concerning burnout evaluation are urgently required to detect burnout of physician workers. Before the pandemic, 39% of physicians reported depression in the USA, with about 400 physicians’ suicides per year, which is twice the rate of the general population [46]. In a study conducted on HCWs in New York, Singh et al. [47] observed that physicians redeployed to treat COVID-19 patients had higher reported emotional exhaustion. Comparable findings have been reported in the European context, where the burnout rate was significantly increased due to the COVID-19 pandemic [48, 49].

Building on previous Italian adaptation on the general population [31] and an Italian teachers’ sample [30], we expected to confirm earlier findings regarding the BAT’s factorial validity, reliability, and construct validity with a sample of HCWs. Details about the results are discussed below.

Concerning the factor validity, we expected the BATxHCW to confirm the second-factor structure and show a good fit for the first-order six-factor structure, following previous validations [30, 31]. Therefore, a CFA was conducted, using RMSEA, SRMR, CFI, and TLI as fit indexes. Findings showed an excellent fit, thus confirming Schaufeli et al. [29] positions, according to which the second-order factor model (by distinguishing between core and secondary symptoms) allows for a valuable theoretical description of the burnout construct, as well as for the recognition of burnout as a syndrome (namely, a constellation of symptoms). Furthermore, apart from confirming previous validations with different Italian samples, our data align with previous confirmation investigations from other European and Japanese studies [50, 51].

We expected to confirm the good reliability already showed by previous validations on different samples concerning the reliability. Consistently, Cronbach’s alpha and McDonald’s omega related to the BAT subscales and the second-order factors proved satisfactory internal reliability of the scale. Indeed, according to George and Mallery’s cut-off values, all the obtained scores ranged from good to excellent, except for the subscale of Psychosomatic Complaints, which showed an acceptable level of internal consistency (α = 0.72).

Finally, concerning internal and external validity, we expected to observe a positive association with the emotional exhaustion subscale of the MBI and negative correlations with the World Health Organization Well-Being Index (WHO-5) and the SF-12 Health Survey (SF-12). Again, Pearson’s correlations confirmed our expectations.

Overall, the present study evaluates the factorial validity, reliability, and construct validity of the BAT in the Italian context and regarding the experience of healthcare workers. However, to the best of our knowledge, despite several pieces of evidence on the saliency of burnout risk for helping professionals in the healthcare sector [29], the BAT measure has not been validated on healthcare professionals in other countries. At the same time, a study on healthcare professionals working in the palliative sector [52] showed that BAT is a reliable tool to assess the risk for burnout in this type of profession. Together with Dijxhoorn and colleagues’ ones, our findings support the multifaceted nature of the burnout construct.

Overall, in a pandemic situation, the current study may support operators in estimating the psychological well-being of health care workers. The ever-increasing number of confirmed and suspected cases of Covid-19, overwhelming workload, depletion of personal protection equipment, widespread media coverage, lack of specific drugs, and feelings of being inadequately supported may all contribute to the mental burden of these HCWs [53]. In this regard, HCWs’ burnout might express the long-term health impacts of the COVID-19 pandemic.

## Limitations and future directions

Our contribution is not without limitations. Firstly, the professionals included in the sample have been faced (and are still facing) a challenging period at work that mirrors the social strain due to the pandemic. Therefore, some findings may be conditioned by the unprecedented times related to the covid pandemic that increased the job demands of HCW and posed new challenges. Secondly, the sample was not gender-balanced and mainly was composed of physicians and nurses, which could have affected the results and their generalizability. While the sample composition is due to a convenience sampling method, further studies may encompass balanced samples regarding gender and HCW roles. A more balanced sample would allow to test the BAT invariance for age and gender [50] and give greater detail on the burnout profiles of HCW.

Overall, the new adaptation of the tools for HCWs could support comparative studies focusing on how different occupational settings impact burnout risk. Specifically, the BAT's multi-dimensionality may lead to deeply investigating burnout syndromes among several working environments. In this regard, the BAT may overcome the existing measures of burnout more focused on emotional dimensions rather than cognitive and psychosomatic ones.

## Conclusions

The current study addressed the validation of a new tool for evaluating burnout symptoms in the healthcare workers in the Italian context. The questionnaire, previously validated for teachers, now, through findings achieved in the present study, is available for healthcare workers. The BATxHCW is a good measure for future studies with a more representative sample.

Furthermore, it is a promising tool for comparative studies within and among countries. Thanks to its good convergent and discriminant validity, the BATxHCW is a valuable instrument to be used in association with other measures able to evaluate the psychological well-being measures of HCW.

Due to the critical role of HCW’s well-being dimensions in the quality of hospital services, future research should further address this topic by validating and using instruments useful for prevention programs.

## Data Availability

This published article includes all the data generated or analyzed during the study. The raw data of this research can be obtained from contacting the authors.

## Abbreviations

HCWs: Healthcare workers
BAT: Burnout Assessment Tool
MBI: Maslach Burnout Inventory
MBI-GS: Maslach Burnout Inventory-General Survey
PD: Psychological distress
PC: Psychosomatic complaints
PC EE: Emotional exhaustion
DP: Depersonalization
PA: Personal accomplishment
WHO-5: World Health Organization Well-Being Index
SF-12: Health Survey Short Form
CFA: Confirmatory factor analysis
RMSEA: Root Mean Square Error of Approximation
CFI: Comparative Fit Index
TLI: Tucker Lewis Index
SRMS: Standardized Root Mean Square Residual
